# Statin adherence improves with age and subsequent treatment sequences: A retrospective cohort study using Proportion of Days Covered (PDC)

**DOI:** 10.1371/journal.pone.0325293

**Published:** 2025-06-25

**Authors:** Aleš Tichopád, Gleb Donin, Jan Žigmond, Jakub Ráfl, Marian Rybář, Petra Šedová, Michal Vrablík

**Affiliations:** 1 Department of Biomedical Technology, Faculty of Biomedical Engineering, Czech Technical University, Prague, Czech Republic; 2 Department of Internal Medicine and Cardiology, University Hospital Brno and Faculty of Medicine, Masaryk University, Brno, Czech Republic; 3 Department of Neurology, International Clinical Research Center, St. Anne’s University Hospital and Faculty of Medicine, Masaryk University, Brno, Czech Republic; 4 Department of Neurology, Mayo Clinic, Rochester, Minnesota, United States of America; 5 3rd Department of Medicine, General University Hospital and First Faculty of Medicine, Charles University, Prague, Czech Republic; University of New South Wales, AUSTRALIA

## Abstract

**Background:**

Dyslipidaemia is a key risk factor for atherosclerotic cardiovascular disease (ASCVD), necessitating effective statin therapy. Despite statins’ proven safety and efficacy, adherence remains suboptimal, with significant gaps between clinical practice and guideline recommendations.

**Methods:**

This retrospective cohort study analysed anonymized health administrative claims data from six employee health funds in the Czech Republic, covering approximately 40% of the insured population from January 1, 2017, to December 31, 2020. We identified statin-incident as well as prevalent cohort of patients. Adherence to statin therapy was assessed using the proportion of days covered (PDC) metric, with factors such as age, gender, sequence of use, and treatment intensity considered as modifiers.

**Results:**

Among the statin-prevalent cohort (SP, n = 890,180), 83.5% achieved a PDC ≥ 50%, and 61.0% reached a PDC ≥ 80%. In the statin-incident cohort (SI, n = 287,871), a clear trend of increasing adherence with age and medication sequence was observed: in adults aged 18–39 median PDC rose from 84.1% (IQR: 57–100) in the first to 94.7% (IQR: 75.6–100) in the third sequence; in those aged 80 + median PDC rose from 95.0% (IQR: 68.9–100) in the first to 100% (IQR: 78.3–100) in the third sequence. Logistic regression identified age (OR=1.011 per year), female gender (OR=0.896), high-intensity treatment (OR=0.975), and second (OR=1.267) or later treatment sequences (OR=1.704) as significant predictors of adherence (all p < 0.001).

**Conclusion:**

Adherence to statin therapy improves with subsequent treatment sequences and age. These findings highlight the need for targeted interventions to enhance adherence, particularly among younger patients. The PDC metric is recommended for integration into clinical practice to monitor and improve medication adherence.

## Introduction

Dyslipidemia is a key modifiable risk factor for atherosclerotic cardiovascular diseases (ASCVD), with statins serving as the cornerstone of lipid-lowering therapy. Despite their proven efficacy and safety [1], the DA-VINCI study revealed that only 33% of patients met their risk-based goals according to 2019 ESC/ EAS guidelines [2]. In Central and Eastern Europe, goal attainment was even lower, with only 24% reaching the recommended LDL-C goal levels – a highlighting a persistent gap between clinical practice and guideline recommendations [3]. In addition to the well-documented, relatively low utilization of maximum-intensity statin monotherapy and reluctance to utilise combinations instead of statin monotherapy [2,4], the efficacy of statin therapy significantly relies on patient adherence, which continues to pose a challenge [5–10]. Adherence to statin therapy varies widely across Europe, shaped by patient-related, healthcare system, and socioeconomic factors. Notably, disparities exist both between and within countries—residing in certain regions can lower adherence odds by up to 50% [11]. In Norway, only 10% of individuals with hypercholesterolemia were on lipid-lowering therapy in 2019, and although adherence among users was relatively high (72.2% for ezetimibe, 84.9% for simvastatin), 40% patients had gaps in statin treatment of 180 days or more [12]. In Sweden, suboptimal adherence was particularly evident among older women with immigrant background [13], and low-income individuals [14]. In Lithuania, only 41% of new statin users remained persistent after one year. Factors associated with higher persistence included older age, higher statin doses, and the use of statins for secondary prevention [15]. A recent multinational analysis across 39 European countries and Israel further emphasized the need for targeted, context-specific strategies to improve adherence and reduce the associated health and economic burden [16].

The Proportion of Days Covered (PDC) is a measure that assesses medication adherence by calculating the percentage of days patients had their medication available during a specified period, using prescription refill data [17]. It is considered a more accurate and conservative measure than the Medication Possession Ratio, especially for regimens involving multiple medications, as it accounts for actual days covered rather than just days supplied. PDC is recommended by healthcare organizations for measuring adherence and has been incorporated into quality assessments and plan ratings [18].

This study aimed to analyse the level of adherence to statins, assessed as the PDC, based on administrative claims data in a large dataset of patients from the Czech Republic representing a region relatively less studied so far where similarly robust analysis has not been performed. Factors such as age, gender, the sequence of use, and the intensity of the statin medication were considered as modifiers.

## Materials and methods

### Study design and data source

We requested the Health Insurance Bureau (HIB) to extract patient of the studied cohorts listed below. The HIB performed the extraction and subsequently anonymized the data. The source database of the HIB covered beneficiaries from six out of seven health insurance companies in the Czech Republic between 2017 and 2022, representing approximately 40% of the mandatory insured population of 10,95 million. Given the relatively uniform and highly regulated healthcare reimbursement system in the Czech Republic, this population can be considered representative of the overall insured population. The patient data contained detailed information on the date of prescription of medications, including the drug name, Anatomical Therapeutic Chemical (ATC) code, dose, and package size, as well as patients’ gender and year of birth.

### Study population

The population of interest comprises individuals treated with statins specifically defined as those with the following ATC codes in their available HAC history: simvastatin (C10AA01); fluvastatin (C10AA04); atorvastatin (C10AA05); rosuvastatin (C10AA07); and fixed combinations of simvastatin and ezetimibe (C10BA02); atorvastatin and ezetimibe (C10BA05); rosuvastatin and ezetimibe (C10BA06); atorvastatin and amlodipine (C10BX03); and atorvastatin, amlodipine, and perindopril (C10BX11). For fixed combinations, only the statin dose was considered qualitatively and quantitatively.

### Study cohorts

We defined statin-incident (SI) study cohort as patients who had not received any statin therapy for a verifiable statin-free period of at least 365 days prior to their commencing statin therapy. Hence the date of the first statin claim comprises the index date. The statin-prevalent (SP) cohort included all patients on statin therapy without a verifiable statin-free period of at least 365 days prior to the index date. Therefore, the available follow up period ranged from 2018 to 2022. The statin prevalent cohort had no index date, as it was analysed cross-sectionally, capturing all statin use observed during the period from 2017 to 2022, without a defined start or end of treatment.

### Exclusion criteria and data trimming

To ensure our analysis focused on patients with consistent and well-established therapy, we applied the following exclusions and data trimming:

Patients with only a single medication claim without follow-up were excluded.

Patients with two medications claims for different ATC codes or dosages within a 30-day period without further continuation were excluded.

We trimmed the early phase of medication optimization and considering established treatment as the first instance where at least two subsequent prescriptions were identical in both the ATC code and dosage.

Data stratification and covariatesFor each patient of the SI cohort, we established sequence numbers for each uninterrupted or unchanged consistent statin medication since initiation. The initial uninterrupted regimen was numbered as the first sequence until it interrupted or changed. Changes in ATC code or dosage marked the start of a new sequence. Generic switching, substitution, or compounded dosages of medications with identical ATC codes and dosages were not considered changes.

Patients were divided into four age groups based on their age at the start of medication: “Adults” (18–39 years), “Middle-aged” (40–59 years), “Aged” (60–79 years), and “Aged 80+” (80 years and above).

We defined intensive statin treatment as a sequence with atorvastatin at 40 mg daily or higher and rosuvastatin at 20 mg daily and higher, irrespective of combination with other drugs. Otherwise, treatment was classified as low to moderate intensity. This classification applied only to individual treatment sequences.

### The proportion of days covered

The PDC, expressed as a percentage, was calculated as follows:


$$\begin{array}{c} PDC=\left(\frac{\text{Total\textbackslash{} number\textbackslash{} of\textbackslash{} days\textbackslash{} covered\textbackslash{} by\textbackslash{} the\textbackslash{} medication\textbackslash{} in\textbackslash{} the\textbackslash{} observation\textbackslash{} period}}{\text{Total\textbackslash{} number\textbackslash{} of\textbackslash{} days\textbackslash{} in\textbackslash{} the\textbackslash{} observation\textbackslash{} period}}\right)\times 100 \end{array}$$

Where:

“Total number of days covered by the medication” is the sum of all days the medication was available to the patient, without considering overlap.

“Total number of days in the observation period” is the length of the period during which adherence is measured.

For the SI cohort, PDC was calculated separately for each medication sequence, allowing its clear association with an ATC code and dose. In the SP cohort, overall PDC was calculated by summing total days covered by statin medication and total days in the entire available period.

If a patient refilled prescriptions before depleting the current supply, the start date of the new prescription was extended to the day after the previous supply was expected to run out, ensuring continuity without artificially inflating adherence rates. The maximum PDC was capped at 1.0, acknowledging any remaining supply at the study’s end or upon switching medications.

### Factors of adherence

We conducted logic transformation regression in SAS v.9.4 for Windows to identify predictors of medication adherence, defined as PDC of 80% or higher in the SP cohort. Independent variables included age, sex, treatment intensity (low to moderate vs. intensive), and the medication sequence as the 1^st^, 2^nd^ and later. The analysis aimed to ascertain the impact of these factors on the likelihood of adherence, setting the significance level at α = 0.05.

### Ethics

This study covered by an ethical approval granted for a broader research project focused on the analysis of retrospective administrative data from healthcare payers (ethics committee of General Faculty Hospital in Prague č.j. 61/23 Grant AZV VES 2024 VFN). This study conforms with the guidelines outlined in the Declaration of Helsinki.

## Results

### Patient demographics

The statin-prevalent (SP) cohort comprised 890,180 patients, with 49.27% (438,585) of males and 50.73% (451,596) of females. The age distribution was as follows: Adults (18–39 years) constituted 4.68% (41,631 patients), Middle-aged (40–59 years) made up 43.03% (383,033 patients), Aged (60–79 years) accounted for 44.84% (399,118 patients), and Aged 80+ (80 years and above) represented7.44% (66,207 patients).

The statin-incident (SI) cohort consisted of 287,871 patients, with 49.38% (142,146) males and 50.62% (145,725) females. The age group distribution was as follows: Adults 8.34% (24,008 patients), Middle-aged 51.89% (149,386 patients), Aged 35.82% (103,125 patients), and Aged 80 + 3.94% (11,352 patients).

### Statin utilization

The most prescribed statin in the first treatment sequence is atorvastatin, accounting for 53.4% (22.1% 10 mg + 25.5% 20 mg + 0.4% 30 mg + 4.0% 40 mg + 1.4% 80 mg) of all first-line prescriptions. Rosuvastatin follows as the second most frequently used molecule, making up 40.5% (22.3% 10 mg + 1.1% 15 mg + 14.3% 20 mg + 0.3% 30 mg + 2.4% 40 mg) of first-line prescriptions. Atorvastatin remains the dominant choice in second-line treatment, representing 46.5% (11.4% 10 mg + 21.3% 20 mg + 1.4% 30 mg + 9.8% 40 mg + 2.3% 80 mg) of prescriptions. Rosuvastatin is again the second most frequently used molecule, covering 40.4% (14.2% 10 mg + 1.3% 15 mg + 18.0% 20 mg + 1.3% 30 mg + 5.5% 40 mg) of second-line therapies. Atorvastatin continues to lead in later lines of therapy, constituting 47.5% (10.5% 10 mg + 18.4% 20 mg + 1.5% 30 mg + 11.1% 40 mg + 5.2% 80 mg) of prescriptions, followed by rosuvastatin at 36.5% (11.4% 10 mg + 1.4% 15 mg + 14.9% 20 mg + 1.6% 30 mg + 7.2% 40 mg). A small proportion of patients transition to adjunctive lipid-lowering therapies, such as ezetimibe (C10BA02, 0.3% first-line, 1.7% second-line) or PCSK9 inhibitors like alirocumab (C10BX11), which appears in 2.4% of first-line and 3.9% of second-line prescriptions. (Supplement S1 and S2 Tables).

### Overall Proportion of Days Covered (PDC)

**Fig 1:**
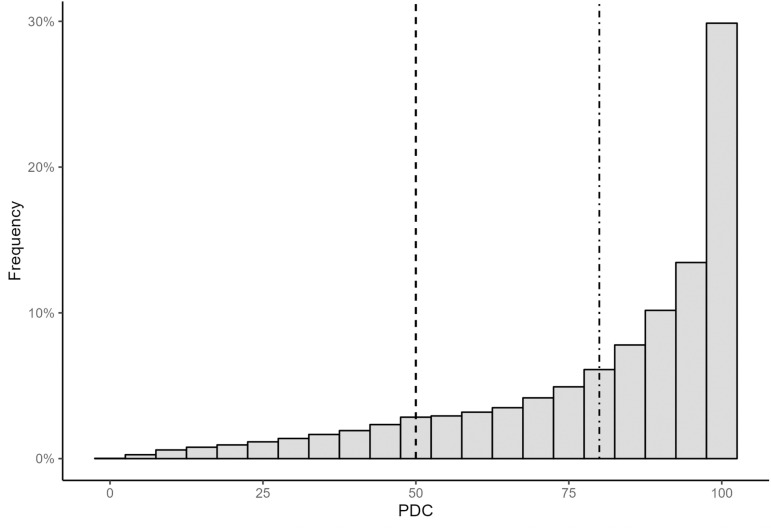
Statin-prevalent cohort: Distribution of the overall proportion of days covered (PDC) aggregate over the study period in the of patients treated with statins.

In the SP cohort, 12,46% of patients failed to achieve PDC of at least 50% and, notably, only 64.55% reached a PDC of 80% or higher, a common adherence threshold (Fig 1).

Similarly, for the first statin sequence in the SI cohort, 13,79% of patients failed to achieve a PDC of at least 50%, while 65.13% of patients had PDC of 80% or more.

**Fig 2:**
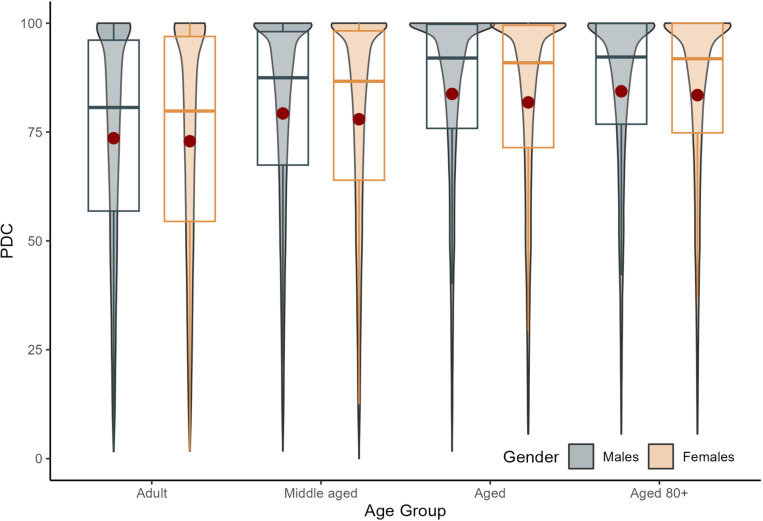
Statin-prevalent cohort: Overall proportion of days covered (PDC) by age group and gender as violin plot with density indicated by the width of the needle. Adults (18–39 years), Middle aged (40–59 years), Aged (60–79 years), and Aged 80+ (80 years and above).

Fig 2 illustrates the overall PDC by age and sex groups, revealing a noticeable trend of increasing PDC with age among statin-prevalent patients. The oldest cohort (Aged 80+) exhibited the highest adherence levels, indicating a stronger propensity towards consistent medication use in this group. Sex appeared to have a lower impact on adherence, as evidenced by the slightly overlapping distributions between male and female patients within each age category.

The median PDCs with interquartile ranges (IQR) for the Adult, Middle-aged, Aged, and Aged 80 + groups, were as follows: Adult males and females had medians of 80.65 (IQR: 56.85–96.10) and 79.82 (IQR: 54.47–96.95), respectively. Middle-aged males and females had medians of 87.47 (IQR: 67.42–98.06) and 86.66 (IQR: 63.94–98.23), respectively. Aged males and females had medians of 91.99 (IQR: 75.84–99.75) and 90.91 (IQR: 71.43–99.50), respectively. Males and females Aged 80 + had median PDCs of 92.23 (IQR: 76.80–99.94) and 91.84 (IQR: 74.81–100.00), respectively (Table 1).

**Table 1:** Statin-prevalent cohort: The PDC medians with interquartile range across age groups and by gender.

Age Group	Males (Median [IQR])	Females (Median [IQR])
Overall	89.52 [70.59-98.90]	89.29 [68.16-99.03]
Adult	80.65 [56.85-96.10]	79.82 [54.47-96.95]
Middle-aged	87.47 [67.42-98.06]	86.66 [63.94-98.23]
Aged	91.99 [75.84-99.75]	90.91 [71.43-99.50]
Aged 80+	92.23 [76.80-99.94]	91.84 [74.81-100.00]

### PDC by sequence

**Fig 3:**
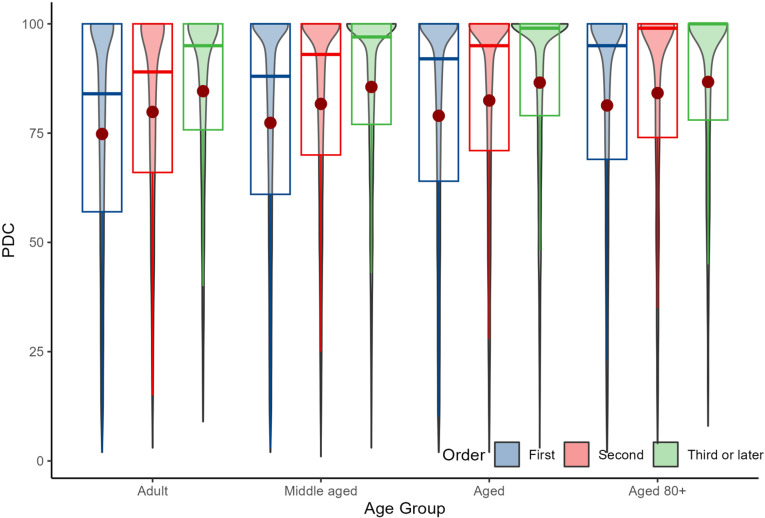
Statin-incident cohort: Proportion of days covered (PDC) by statin sequence and age group as violin plot with density indicated by the width of the needle. Adults (18–39 years), Middle aged (40–59 years), Aged (60–79 years), and Aged 80+ (80 years and above).

Table 2 and Fig 3 display the overall PDC patterns across age groups and different medication sequences in SI patients. The data reveal a trend of increasing aggregated PDC across sequences, particularly in the ‘Adult’ and ‘Aged’ groups, with ‘Adults’ improving from a median PDC of 84.11 to 94.74 and those in the Aged group showing a significant increase from 91.98 to 99.01.

**Table 2:** Statin-incident cohort: The PDC medians with interquartile range across age groups and different statin sequences.

Age Group	Sequence 1 (Median [IQR])	Sequence 2 (Median [IQR])	Sequence 3 (Median [IQR])
Overall	89.63 [61.73-100]	93.75[69.77-100]	98.13 [77.92-100]
Adult	84.11 [56.60-100]	89.11 [66.30-100]	94.74 [75.55-100]
Middle-aged	88.44 [61.22-100]	92.59 [69.59-100]	97.09 [76.92-100]
Aged	91.98 [63.64-100]	95.24 [70.80-100]	99.01 [79.37-100]
Aged 80+	95.04 [68.94-100]	98.90 [74.16-100]	100.00 [78.26-100]

The trend across age groups clearly indicates that older individuals, particularly those in the ‘Aged’ and ‘Aged80+’ categories, demonstrate higher and more consistent medication adherence, with adherence levels peaking in later sequences.

### Factors of adherence

A logistic regression analysis identified several predictors of medication adherence (PDC ≥ 80%) in the SP cohort. Each year increase in age was associated with a 1.1% increase in the odds of achieving at least 80% adherence (OR = 1.011). Females had 10.4% lower odds of adherence compared to males (OR = 0.896). High-intensity statin treatment was associated with a 2.5% reduction in adherence odds (OR = 0.975). Transition to the second sequence of medication increased adherence odds by 26.7% (OR = 1.267) and moving to the third or later sequence improved adherence odds by 70.4% (OR = 1.704). All terms in the model were highly significant with p < 0.001.

The slightly lower adherence observed in females was further evidenced by their aggregate unadjusted median PDC of 89.29, compared to 89.82 for males.

## Discussion

Our study has several important findings identifying medication sequence, age, gender and treatment intensity as significant predictors of adherence to statin therapy. First, adherence to statin therapy tends to improve with each subsequent treatment sequence. This improvement may be attributed to the better tolerability of the newer sequences, as earlier ones might have caused adverse effects [19]. Hence, we suggest that physicians prioritize identifying the most tolerable statin regimen early in treatment to enhance long-term adherence. The analysis of adherence to statin therapy as a function of treatment sequence is a novel approach, which might better reflect the real-world behaviour of statin users. Limiting the impact of the initial phase of finding the best tolerated statin type and dose is crucial to assess the long-term adherence accurately.

Second, adherence rates improved with age, a trend potentially associated with the transition between subsequent medication sequences and a learning curve during early treatment phases [20]. Our findings indicate that older individuals, especially those aged 80 and above, exhibit the highest levels of adherence to statin therapy. The association between age and statin adherence remains inconsistently reported. While some studies indicate that the likelihood of statin non-adherence and statin intolerance (SI) increases with age [21,22], others suggest that older and very elderly patients may demonstrate better adherence compared to younger individuals [11,12,23,24]. Notably, some studies exclude patients over the age of 80, limiting insight into adherence patterns in this growing population group (Norway) [12], Some suggest that older individuals may have a higher awareness or concern for their health, especially concerning cardiovascular risks [5,25]. They might be more committed to following medical advice, including medication regimens, due to their increased risk of cardiovascular events. However, other studies on hypertension have shown that adherence peaks among people aged 60–69 and decreases in those 70 and older [26]. Age may play a crucial role in medication adherence, potentially due to more established routines or better understanding of medication importance among older patients. Conversely, the presence of multimorbidity requiring polypharmacotherapy and the cognitive impairments that accompany aging could negatively influence adherence [27]. Therefore, while older adults may generally show higher adherence rates, the complexities of their health conditions necessitate careful management.

Third, our analysis revealed that women had slightly higher odds of not achieving 80% of adherence to statin therapy compared to males. Some previous studies indicated gender-based disparities in medication adherence, with women generally being less likely to adhere to statin therapy than men, potentially due to differences in side-effect profiles or health beliefs (USA [28], Sweden [13], metaanalysis [29], review [11]). Our findings contribute to the ongoing debate about the role of gender in medication adherence and highlight the need for targeted interventions to improve adherence among patients with dyslipidemias, regardless of sex. These interventions could include patient education, personalized treatment plans, and adherence support programs, which have been shown to be effective in improving medication adherence in various patient populations [20,30,31]. The lower impact of gender on adherence in our study suggests that adherence strategies should be broadly applicable across both genders.

Fourth, high-intensity statin treatment was associated with a slightly lower odds of adherence. However, despite statistical significance, likely driven by the large sample size, there were no clinically meaningful differences in the proportion of days covered (PDC) between statin types or intensity levels. The mean PDC was comparable. Generally, we observed that approximately 65% of patients achieve a PDC of at least 80%, indicating a significant gap in statin adherence within this patient population. This finding aligns with existing literature, which consistently highlights adherence issues in the management of chronic diseases, particularly dyslipidemias [32]. A systematic review from 2014 reported a significantly elevated risk of CVD with a risk estimate assessed between 1.22 and 5.26 and mortality risk estimates assessed between 1.25 and 2.54 among non-adherent individuals [32].

The PDC is a straightforward yet effective measure of adherence, a metric based on a simple and intuitive principle that requires complex calculations but can be easily automated. PDC might be included into electronic health records and calculated automatically allowing physicians to quickly assess whether patients have likely refilled their prescriptions in a timely manner by comparing the dates of prescription receipt and package sizes during consultations. Automated PDC calculations can trigger an alert to discuss medication adherence serving as a simple reminder to clinicians, pharmacists, as well as the patients [17,33].

Digital tools, such smartphones and applications, can enhance medication adherence by keeping patients informed and facilitating communication among healthcare providers, pharmacies, payers, and patients. This interconnected approach aligns incentives across all parties to support patient health outcomes [34].

It is worth noting, that statin discontinuation and non-adherence are most commonly driven by statin-associated adverse effects, misinformation and insufficient patient education, which can lead to fear of side effects. While non-adherence is frequently attributed to patients, physician inertia, also plays a role [35]. A recent systematic review identified modifiable factors influencing adherence, including patients’ beliefs, disease knowledge, environmental and social influences with a strong patient–provider relationship being a key adherence facilitator [36]. Physicians and pharmacists up-to-date clinical knowledge regarding statin therapy plays a key role [37].

Our study has a few limitations. Most importantly, the use of administrative data cannot accurately reflect real medication exposure and, thus, there is a possibility even patients with high PDC may not necessarily use their medications as prescribed. We further assumed that all medications were obtained through prescription fills, and that there were no unrecorded treatment pauses or dosage changes. Our analysis did not account for hospital stays, and we presumed a daily dose of one unit for all medications. This analysis does not allow for the examination of other important factors that may have contributed to non-adherence (e.g., socioeconomic status) [11]. In our study, the TEN SPIDER algorithm [33] was not feasible due to the frequent occurrence of missing data. Additionally, implementing this approach on large datasets is time-consuming and does not appear to substantially improve the accuracy of the results.

## Conclusion

Adherence to statin therapy is a dynamic process influenced by age, gender, treatment sequence, and intensity. While overall adherence improves with age and subsequent therapy adjustments, a substantial proportion of patients still fail to meet the 80% adherence threshold, particularly younger adults. Integrating PDC metrics into electronic health records may facilitate timely interventions. Targeted support—especially for younger patients—and the use of digital tools to engage patients and improve communication across healthcare providers are essential to optimize statin adherence and improve cardiovascular outcomes.

## Supporting information

S1 Table Frequency of lipid-lowering agents used in the first, second, and third or later treatment sequences. (DOCX)

S2 Table Frequency of lipid-lowering agents used in the first, second, and third or later treatment sequences (including doses). (DOCX)
