# Supplementary material for: Statin adherence improves with age and subsequent treatment sequences: A retrospective cohort study using Proportion of Days Covered (PDC)
Source: PLoS One. 2025 Jun 25;20(6):e0325293. doi: 10.1371/journal.pone.0325293 (PMC12193063; doi:10.1371/journal.pone.0325293)
Supplement: S1 Table — (DOCX) [file pone.0325293.s001.docx]

# Table S1. Frequency of lipid-lowering agents used in the first, second, and third or later treatment sequences

| **Molecule** | **First sequence** | **Second sequence** | **Third and later** |
| --- | --- | --- | --- |
| Atorvastatin | 111393 (53.4%) | 23888 (46.5%) | 13337 (47.5%) |
| Rosuvastatin | 84507 (40.5%) | 20723 (40.4%) | 10239 (36.5%) |
| Alirocumab | 5116 (2.5%) | 1989 (3.9%) | 789 (2.8%) |
| Simvastatin | 2445 (1.2%) | 678 (1.3%) | 476 (1.7%) |
| C10BA06 | 1622 (0.8%) | 2183 (4.3%) | 1869 (6.7%) |
| C10BX03 | 1605 (0.8%) | 430 (0.8%) | 183 (0.7%) |
| C10AA04 | 649 (0.3%) | 302 (0.6%) | 208 (0.7%) |
| Ezetimibe + Statin | 587 (0.3%) | 258 (0.5%) | 173 (0.6%) |
| Atorvastatin + Amlodipine | 539 (0.3%) | 885 (1.7%) | 798 (2.8%) |
