# Supplementary material for: Statin adherence improves with age and subsequent treatment sequences: A retrospective cohort study using Proportion of Days Covered (PDC)
Source: PLoS One. 2025 Jun 25;20(6):e0325293. doi: 10.1371/journal.pone.0325293 (PMC12193063; doi:10.1371/journal.pone.0325293)
Supplement: S2 Table — (DOCX) [file pone.0325293.s002.docx]

# **Table S2. Frequency of lipid-lowering agents used in the first, second, and third or later treatment sequences (including doses)**

| **Molecule (Dose)** | **First sequence** | **Second sequence** | **Third and later** |
| --- | --- | --- | --- |
| Atorvastatin (C10AA05 20 mg) | 53237 (25.5%) | 10949 (21.3%) | 5165 (18.4%) |
| Rosuvastatin (C10AA07 10 mg) | 46572 (22.3%) | 7273 (14.2%) | 3190 (11.4%) |
| Atorvastatin (C10AA05 10 mg) | 45982 (22.1%) | 5871 (11.4%) | 2943 (10.5%) |
| Rosuvastatin (C10AA07 20 mg) | 29830 (14.3%) | 9260 (18.0%) | 4185 (14.9%) |
| Atorvastatin (C10AA05 40 mg) | 8314 (4.0%) | 5043 (9.8%) | 3127 (11.1%) |
| Rosuvastatin (C10AA07 40 mg) | 5015 (2.4%) | 2826 (5.5%) | 2009 (7.2%) |
| Alirocumab (C10BX11 20 mg) | 3518 (1.7%) | 1361 (2.7%) | 484 (1.7%) |
| Atorvastatin (C10AA05 80 mg) | 2966 (1.4%) | 1183 (2.3%) | 1471 (5.2%) |
| Rosuvastatin (C10AA07 15 mg) | 2337 (1.1%) | 684 (1.3%) | 385 (1.4%) |
| Simvastatin (C10AA01 20 mg) | 1591 (0.8%) | 400 (0.8%) | 230 (0.8%) |
| Alirocumab (C10BX11 10 mg) | 1510 (0.7%) | 503 (1.0%) | 162 (0.6%) |
| Atorvastatin (C10AA05 30 mg) | 844 (0.4%) | 713 (1.4%) | 428 (1.5%) |
| C10BA06 (C10BA06 10 mg) | 829 (0.4%) | 596 (1.2%) | 415 (1.5%) |
| Rosuvastatin (C10AA07 30 mg) | 726 (0.3%) | 648 (1.3%) | 449 (1.6%) |
| C10BA06 (C10BA06 20 mg) | 707 (0.3%) | 1149 (2.2%) | 872 (3.1%) |
| Simvastatin (C10AA01 10 mg) | 688 (0.3%) | 222 (0.4%) | 169 (0.6%) |
| C10AA04 (C10AA04 80 mg) | 649 (0.3%) | 302 (0.6%) | 208 (0.7%) |
| C10BX03 (C10BX03 5 mg) | 596 (0.3%) | 96 (0.2%) | 44 (0.2%) |
| Ezetimibe + Statin (C10BA02 10 mg) | 587 (0.3%) | 258 (0.5%) | 173 (0.6%) |
| C10BX03 (C10BX03 10 mg) | 570 (0.3%) | 153 (0.3%) | 61 (0.2%) |
| Atorvastatin + Amlodipine (C10BA05 10 mg) | 539 (0.3%) | 885 (1.7%) | 798 (2.8%) |
| C10BX03 (C10BX03 20 mg) | 439 (0.2%) | 181 (0.4%) | 78 (0.3%) |
| Simvastatin (C10AA01 40 mg) | 166 (0.1%) | 54 (0.1%) | 76 (0.3%) |
| Alirocumab (C10BX11 40 mg) | 88 (0.0%) | 125 (0.2%) | 141 (0.5%) |
| C10BA06 (C10BA06 40 mg) | 75 (0.0%) | 431 (0.8%) | 581 (2.1%) |
| Atorvastatin (C10AA05 60 mg) | 45 (0.0%) | 107 (0.2%) | 171 (0.6%) |
| Rosuvastatin (C10AA07 5 mg) | 22 (0.0%) | 5 (0.0%) |  |
| C10BA06 (C10BA06 5 mg) | 11 (0.0%) | 6 (0.0%) | 1 (0.0%) |
| Atorvastatin (C10AA05 120 mg) | 4 (0.0%) | 14 (0.0%) | 19 (0.1%) |
| Rosuvastatin (C10AA07 60 mg) | 2 (0.0%) | 16 (0.0%) | 11 (0.0%) |
| Rosuvastatin (C10AA07 45 mg) | 2 (0.0%) | 6 (0.0%) | 4 (0.0%) |
| Atorvastatin (C10AA05 100 mg) | 1 (0.0%) | 1 (0.0%) | 5 (0.0%) |
| Rosuvastatin (C10AA07 50 mg) | 1 (0.0%) | 2 (0.0%) | 5 (0.0%) |
| Simvastatin (C10AA01 30 mg) |  | 1 (0.0%) | 1 (0.0%) |
| Atorvastatin (C10AA05 90 mg) |  | 3 (0.0%) | 5 (0.0%) |
| C10BA06 (C10BA06 30 mg) |  | 1 (0.0%) |  |
| Simvastatin (C10AA01 60 mg) |  | 1 (0.0%) |  |
| Atorvastatin (C10AA05 70 mg) |  | 1 (0.0%) |  |
| Rosuvastatin (C10AA07 70 mg) |  | 1 (0.0%) |  |
| Rosuvastatin (C10AA07 25 mg) |  | 2 (0.0%) | 1 (0.0%) |
| Atorvastatin (C10AA05 50 mg) |  | 3 (0.0%) | 3 (0.0%) |
| Alirocumab (C10BX11 60 mg) |  |  | 2 (0.0%) |
